# T-wave Oversensing with Inappropriate Therapy in Remote Monitoring

**Published:** 2010-06-05

**Authors:** Manuel Frutos, Alonso Pedrote, Eduardo Arana, Juan Sanchez-Brotons

**Affiliations:** Arrhythmia Unit, Cardiology department, Hospitales Universitarios Virgen del Rocio, Seville, Spain

**Keywords:** Remote monitoring, T-wave oversensing, implantable cardioverter-defibrillator

## Abstract

T-wave oversensing can cause inappropriate implantable cardioverter-defibrillator (ICD) therapies that are difficult to correct. Remote monitoring allows follow-up of ICD patients without visiting the hospital and can help in early detection of any malfunctions. We describe the case of a patient who experienced inappropriate antitachycardia pacing therapy due to T-wave oversensing; the problem was promptly detected by remote monitoring and corrected by device reprogramming.

## Introduction

Inappropriate shocks are an important disadvantage of implantable cardioverter-defibrillators (ICD) [1]. Following supraventricular arrhythmias, inappropriate device sensing is the most common cause of inappropriate shocks [2]. Remote monitoring has lessened the workload entailed in ICD follow-up and is helpful for early detection of malfunctions. We describe a case of inappropriate ICD therapy caused by T-wave oversensing not due to lead malfunction neither clinical causes that was promptly detected by remote monitoring, which allowed rapid correction of the problem by device reprogramming.

## Case Report

A 43-year-old man with chronic ischemic heart disease, ejection fraction of 22%, in NYHA functional class II, and with a narrow QRS, was referred to our department for implantation of an ICD in primary prevention. A single-chamber device (Virtuoso VR, Medtronic, Minneapolis, Minnesota, USA) with an active-fixation lead (Sprint Quattro Secure™ 6947, Medtronic, Minneapolis, Minnesota, USA) was implanted in the right ventricular apex. At the time of implantation, ventricular sensing with a stable intrinsic amplitude of 9 mV without T-wave sensing was achieved. A single zone of detection-therapy at 188 bpm was programmed, with antitachycardia pacing (ATP) during charging and shock at 35 J, as well as a sensitivity threshold of 0.3 mV. All functional and lead integrity parameters were normal before hospital discharge and at an in-clinic follow-up visit 1 month later. Subsequent follow-up was performed by remote monitoring (CareLink, Medtronic, Minnesota, Minneapolis, USA). The automatic notification alarms were enabled; these alarms were sent to our e-mail address whenever the system detected impedances outside the programmed range, ventricular intervals less than 130 ms (Lead Integrity Alert™ algorithm, Medtronic, Minneapolis, Minnesota, USA), a battery voltage indicating elective replacement, or antitachycardia therapy.

**Figure 1:**
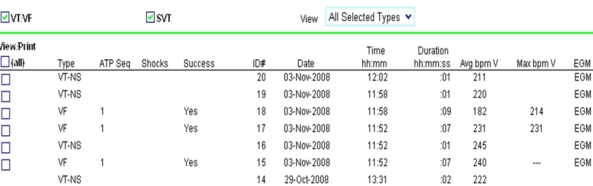
Detailed view of episodes recorded on the patient transmission website. It shows the seven episodes, heart rate sensed, therapy applied and success.

**Figure 2:**
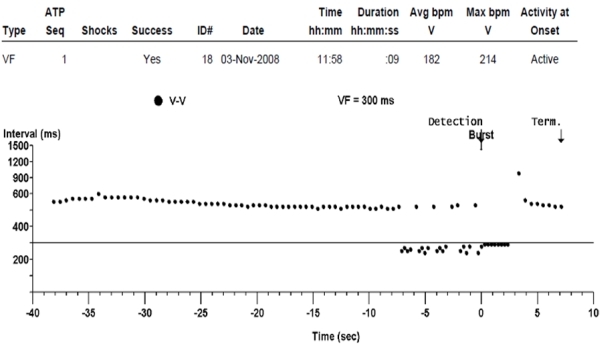
Detection and therapy diagram shown in a graph of intervals between ventricular sensing events. After normal sensed sinus rhythm, the device begins to sense events in ventricular fibrillation zone in a sustained-episode fashion, so it applies therapy (ATP) that finishes the episode.

**Figure 3:**
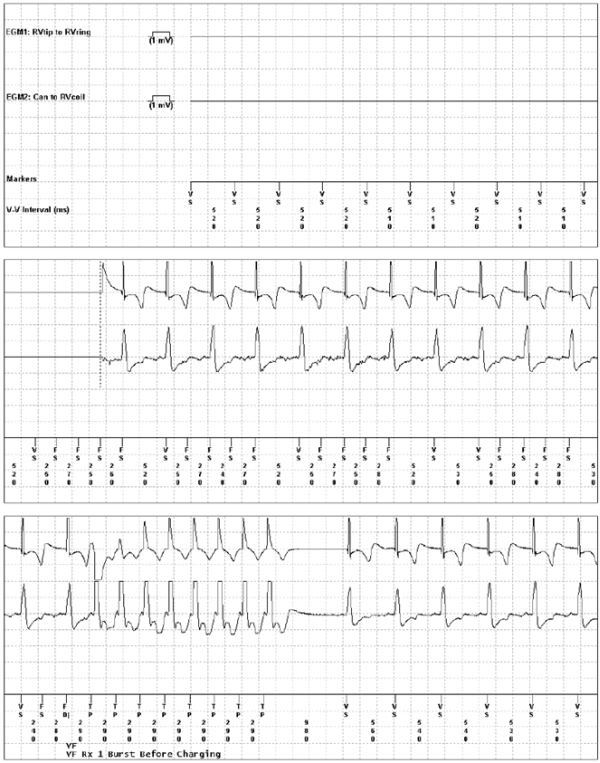
Bipolar (top) and unipolar (middle) intracardiac electrograms and marker channel and intervals (ms) for one of the events. Note the intermittent nature of T-wave sensing and how it disappears after ATP therapy during charging, thereby inhibiting the shock.

Two months post-implant, we received a therapy alert that reported 7 episodes of tachyarrhythmia in the programmed area (Figure 1); 4 were classified as nonsustained and 3 as sustained. All episodes were treated according to the programmed parameters with ATP during charging (Figure 2), which was not noticed by the patient. A review of stored electrograms showed that the episodes were wrongly classified as ventricular fibrillation and were actually T-wave oversensing during normal sinus rhythm (Figure 3). After ATP, no shocks were delivered because T-wave oversensing disappeared during redetection. The patient was notified to come for an in-clinic check-up. No abnormal data related to device operation or lead integrity were observed, and the patient reported no symptoms that suggested myocardial ischemia or heart failure, neither electrolytic alterations nor long QT interval. The device was reprogrammed by increasing the sensitivity threshold to 0.6 mV. No other events were observed 12 months after follow-up.

## Discussion

The ICD has clearly proven its efficacy in the prevention of sudden death secondary to ventricular arrhythmia in high-risk patients. However, up to 15% of patients experience inappropriate shocks during the first year [1]. In 20%, the shocks are secondary to inadequate device sensing [2]. T-wave sensing is the most common cause of ventricular oversensing and representing 14% of such cases [3]. Although the cause was not identified in all patients, Brugada syndrome, long QT syndrome, and hypertrophic cardiomyopathy have been implicated in some cases. Reversible causes are water-electrolyte imbalances and acute myocardial ischemia, and their correction is the first step to attempt to solve the problem. Nevertheless, high T-wave sensing counts are usually hard to resolve and believed to be related to a technical failure in device sensing [4]. In most published cases, it was necessary to reposition the implanted lead, insert an additional lead for sensing, apart from the high power circuit, or implant a new generator. Noninvasive resolution through device reprogramming, as in our patient (who required a simple adjustment in the sensitivity threshold), is unusual.

The number of ICD implants has risen recently due to the results of new studies, but has also increased the health care burden in terms of follow-up. ICD remote monitoring has proven to be extremely useful in routine patient follow-up, as it allows device malfunctions and arrhythmic events to be detected early through web-based alarms [5]. However, to our knowledge, there have been no previous reports of ICD malfunction due to T-wave oversensing with inappropriate therapy not perceived by the patient (ATP), in which the malfunction was promptly detected and corrected thanks to remote monitoring. The web-alert we recived informing us about an antitachycardia therapy, allowed us to analyze the episode and notify the patient to come for prompt device reprogramming, a measure that successfully corrected the problem and prevented new episodes that could have led to inappropriate shocks.
